# Supplementary material for: Are carbon emissions trading and green financial instruments synergistic? -Comprehensive quantitative research based on content analysis
Source: PLoS One. 2024 Mar 7;19(3):e0298601. doi: 10.1371/journal.pone.0298601 (PMC10919601; doi:10.1371/journal.pone.0298601)
Supplement: S1 Data — (ZIP) [file pone.0298601.s001.zip › Data/地级市Z_F工作报告.zip!/║╙─╧/╓▄┐┌╩╨/└┤╫╘╣1⁄2╓┌║┼í╛┬φ┐╦╩2╛▌═°í┐_2016.docx]

各位代表：

　　现在，我代表市人民政府，向大会报告工作，请予审议，并请各位政协委员和其他列席人士提出意见。

　　一、2015年和“十二五”工作回顾

　　2015年，是“十二五”的收官之年，也是我们积极作为、取得显著成绩的一年。一年来，在省委、省政府和市委的正确领导下，市人民政府深入实施周口崛起方略，坚持调中求进，改中创新，转中向好，稳中有为，坚定不移把稳增长、保态势作为事关全局的突出任务，克难攻坚，锐意进取，经济社会发展保持了平稳健康的良好态势。

　　初步核算，全市生产总值增长9%，高于全省0.7个百分点；固定资产投资增长16.9%，社会消费品零售总额增长12.8%，一般公共预算收入增长10.4%，一般公共预算支出增长15.4％，城镇居民人均可支配收入增长9.2％，农村居民人均可支配收入增长9.9％。主要经济指标增速持续高于全省平均水平，较好完成了市三届人大六次会议确定的各项目标任务。

　　一年来，我们主要做了以下工作：

　　（一）坚持抓项目、扩投资，基础支撑继续巩固，发展优势逐步拓展。始终把项目建设作为扩大投资的重要抓手，围绕以“双十五”工程和中心城区“四个一批”为核心的省、市重点项目，不断完善中心城区重点项目协调推进周例会、县市重点项目协调推进月例会工作机制，组织开展71个重点项目集中开工、重点项目“百日会战”和重点项目观摩点评活动，193个市级以上重点项目完成投资662.8亿元，占年度投资计划的109.2%，保持了项目建设的强劲势头。一批重大项目进展顺利，新周高速公路建成通车，郑合高铁开工建设，荷花市场依法关闭搬迁，华耀城一期正式营业，中原鞋城完成规划设计，隆达2×66万千瓦燃煤机组扩建加快实施，沙颍河上游通航工程及港口建设扎实推进。在我市的积极谋划争取下，周口至南阳高速公路、濮阳经周口至潢川铁路、周口通用机场等一批重大基础设施项目列入省重点建设规划。这些大项目的实施，既为当年经济发展提供了动力，也将为未来发展提供基础支撑。

　　（二）坚持抓产业、优结构，质量效益继续提高，经济发展态势良好。着力做优农业、做强工业、做大服务业，经济总量持续壮大，产业结构日趋优化，二三产业占比达到78.2%，质量和效益得到新的提升。农业现代化建设有效推进。新建高标准粮田136万亩，周麦30号、周豆23号顺利通过国审，科技对农业的贡献力度进一步加大，粮食总产达到165.9亿斤，再创历史新高。农业产业化集群发展势头良好，培育国家级农业产业化重点龙头企业5家、省级51家，龙头企业实现销售收入400多亿元。新增土地流转面积17.3万亩、市级以上农民示范合作社71家、工商登记的家庭农场1572家。畜牧、林果、蔬菜等产量、产值继续提高，农业综合效益进一步提升。工业主导作用愈加突出。规模以上工业增加值增长10.7%，增速居全省第一位；实现利润430.5亿元，高于全省平均增速12.4个百分点，居全省第二位。产业集聚区规模以上工业增加值增长17.3%，居全省第三位。3家企业在新三板挂牌，认定6家省级“两化”融合示范企业，完成114个重点技术改造项目。服务业成长势头良好。全市服务业完成增加值658亿元，增长11.3%。商务中心区和特色商业区累计建成面积8平方公里，完成固定资产投资138亿元；周口港区成功创建为省级示范物流园区。新增5家村镇银行，2家农信社具备组建农商行条件。房地产市场下滑势头得到一定遏制，市场逐步回暖。文化旅游产业稳步发展，创建省级文化产业示范基地6个，项城汝阳刘笔业文化产业园被命名为国家级文化产业示范基地；全年接待游客1950万人次，总收入92亿元。

　　（三）坚持抓带动、强辐射，城乡设施继续完善，城镇化水平不断提升。毫不动摇实施中心城区带动战略，切实提高城市建设管理水平，增强城市综合承载和辐射带动能力，城镇化水平得到新的提高。规划编制扎实推进。初步完成《周口市城市总体规划（2014～2030）》成果编制和港口物流产业聚集区、商务中心区、沙北片区、沙南片区等10余项控制性详细规划，中心城区近期建设用地实现控规全覆盖。城市道路、绿地、供水、排水等10余项专项规划付诸实施。中心城区建设管理水平持续提高。八一路、中州大道、宁洛高速出入口综合改造全部完成，太清路、汉阳北路升级改造稳步推进，武盛大道建成启用，新周高速连接线建成通车。新增280台公交车，建设50个公益自行车服务点，新建、改建3个健身园和2个健身广场。完成老水厂日供水5万吨提升改造工程，完成老城区13条主次干道供水管网改造。新建垃圾中转站10座，购置垃圾清运车16辆，城市公共服务能力和水平进一步提升。坚持中心城区城管委月例会制度，深入开展“脏、乱、差、堵”专项治理，扎实推进“交通秩序综合整治”百日行动，保持严厉打击“两违”的高压态势，打造了一批严管街、示范路，城市管理的长效化、精细化、规范化机制正在逐步形成。城乡基础设施日臻完善。周商大道完成改造升级、周项快速通道加快建设，中心城区与四个组团县市之间联系更加紧密。加强路网、电网、气网、通信网建设，“组团县城”与“支撑县城”之间的基础设施进一步完善。推进城市基础设施向农村延伸，新改建农村公路1285公里，完成危桥改造3064延米，光缆通乡、通村工程扎实推进，新解决81万农村居民和26.9万农村师生饮水安全问题，农村生产生活条件明显改善。城镇化水平不断提高。支持县城加快开发建设，提高人口和产业承载能力，县城规模持续扩大，基础设施持续完善，服务功能持续增强，管理水平持续提高，愈发成为宜居、宜业、宜学的县域政治经济文化中心。加快中心城镇建设步伐，沈丘县付井镇、淮阳县四通镇、西华县逍遥镇、项城市秣陵镇、商水县固墙镇、太康县老冢镇等创建为全省第一批重点建设示范镇。落实各类鼓励农民进城落户的政策措施，推进农村人口就近市民化，全市城镇化率达到37.8%，比上年提高1.6个百分点。

　　（四）坚持抓改革、促开放，招商引资继续推进，创新创业活力显著增强。坚持以改革破难题，以创新求突破，改变招商方式，注重招商实效，发展的动力和活力显著增强。改革创新不断深化。积极推动政府职能转变，行政审批效率继续提高。全面推行商事制度改革，促进大众创业、万众创新，全市新登记市场主体4.5万户，增长9.8%，落实小微企业贷款220亿元，增长9.3%。政府机构改革、公务用车制度改革有序推进，交通执法体制改革、食品药品监管体制改革、不动产统一登记改革顺利完成。推进政府和社会资本合作（PPP），吸引社会资本76亿元。大力支持科技创新、业态创新、经营方式创新，新认定国家高新技术企业13家，周口师范学院大学科技园被认定为省级大学科技园，周口国家级农业科技园获科技部批复，郸城巨鑫生物科技公司设立院士科研工作站，农科院国家博士后科研工作站成功获批，西华县电子商务产业园建成开园，扶沟“互联网 蔬菜”销售平台正式上线，商水智慧农业平台、亿星黄淮农产品网开通运营。开放招商成效明显。围绕食品加工、纺织服装、医药化工、电缆电气、装备制造和新型建材六大产业集群，建链、延链、补链、强链，全市新签约招商引资项目123个，签约金额887.6亿元。完成与北京华赢集团合作洽谈，成功引进总投资100亿元以上的世通巴铁研发生产项目。实施亿元以上项目168个，实际到位资金233.4亿元；新批外商投资企业14家，实际利用外资4.9亿美元。要素保障持续加强。积极推进政银企合作，平安银行郑州分行、中原银行分别向我市提供100亿元以上的综合授信。与金融机构合作，建立企业还贷周转资金和小微企业互助贷款风险补偿担保基金，企业融资难逐步得到缓解。银行业新增存贷比继续提高，信贷资金供给有效改善。积极推进与省水投集团、省国土中心合作，探索了中心城区重大基础设施和生态水系建设融资新模式。加强土地、电力、劳动力等资源调配，重点项目、重点产业的建设发展需求得到较好保障。

　　（五）坚持抓保障、促共享，民生福祉继续改善，社会事业全面进步。坚持以人民为中心的发展理念，发展依靠人民，发展成果由人民共享，人民群众有了更多获得感。实施精准扶贫。建立定点帮扶机制，为19.5万贫困户落实了帮扶责任人。完成整村推进项目219个，到户增收试点项目344个，“雨露计划”培训8.1万人次，又有20万人实现稳定脱贫。着力促进就业。新增农村劳动力转移就业10.5万人，新增城镇就业8.8万人，城镇登记失业率控制在3.9%以内，超额完成省定目标任务，完善社会保障。保障性住房建设完成省定目标任务，农村危房改造完成31700户；建成100所农村养老院、5所中心城区社区养老服务示范点；为59万城乡低保对象发放救助金9.4亿元，初步实现了人人享有社会保障的目标。全市新农合参合人数886万人，基本公共卫生服务人均补助标准进一步提高，27家县级公立医院全部实施药品零差率销售。社会事业全面进步。推动义务教育均衡发展，中心城区“三校五园”和县市农村薄弱学校改造项目进展顺利，中心城区新增学位4000多个，“大班额”“入园难”问题逐步缓解。新招聘教师4700人，教师年龄结构、学科结构得到优化。76名考生被清华大学、北京大学录取，河南省高考文理科状元均出自周口；周口师范学院转型发展取得新的进步，周口职业技术学院被授予“河南省职业教育品牌示范学院”称号，周口科技职业学院晋升本科通过专家组初步评估验收，周口技工学校成功获批为“高级技工学校”。医药卫生计生体制改革稳步实施，城乡医疗资源持续增加，医疗和疾病防控能力不断提高，人口发展更加均衡，人民健康更有保障。文化惠民政策全面落实，完成周末公益剧场演出50场，为市图书馆新购图书5万册，公共文化场馆免费开放。获省级以上优秀文艺作品表彰79项。在国家博物馆成功举办“大道周口”王学岭诗文书作展。中国（淮阳）非物质文化遗产展演规模和影响不断扩大。扎实推进文明周口建设，持续开展“践行价值观、文明我先行”主题系列活动和道德模范评选，评选出“文明市民”、“最美教师”100名，国家、省、市级道德模范及提名奖等59名。顺利通过国家二类城市语言文字工作评估验收。成功举办省第十二届老年人体育健身大会和第二届中华龙舟赛，全民健身计划深入实施。生态建设成效明显。持续实施蓝天、碧水、乡村清洁工程，生态环境保持良好。完成省定淘汰黄标车目标任务，严禁农作物秸秆焚烧，加强扬尘治理，空气质量优良天数累计191天，超额完成省定目标任务。加强重点流域河流污染综合治理，地下水饮用水源地取水水质稳定达标。美丽乡村建设稳步推进。完成造林11.9万亩，并实现了高速公路廊道绿化全覆盖。社会治理得到加强。严厉打击各类违法犯罪活动，实现命案破案率100％，群众的安全感和满意度进一步提高；持续开展食品药品安全专项治理，严格落实安全生产责任，全年没有发生重特大事故；依法解决信访诉求，赴京到省来市信访总量平均下降50％以上；深化社会治安综合治理，法治周口、平安周口建设扎实推进，社会大局和谐稳定。

　　一年来，市人民政府办理人大代表建议136件、政协提案399件，办结率100%。自觉加强政府自身建设，深入开展“三严三实”专题教育和“三查三保”活动，深化政务公开，法治政府、服务型政府建设继续推进。严格落实中央“八项规定”和省委、省政府20条意见，自觉履行“一岗双责”，政府廉政工作取得新的成效。国防动员和双拥共建深入开展，军政军民团结更加巩固。妇女儿童、残疾人、红十字、慈善、老龄等事业健康发展，统计、审计、民族、宗教、外事、侨务、史志、档案、气象、地震、社会科学、人民防空、对口援疆等工作取得新的成绩。

　　各位代表！

　　2015年工作任务的全面完成，标志着我们实现了“十二五”的圆满收官，同时也标志着我们已经站在了新的发展起点上。回顾这五年的奋斗岁月，自强不息的千万周口儿女，在充满希望的这方热土上，以顽强的拼搏和辛勤的汗水，用勤劳的双手和聪明的智慧，创造了精彩业绩，谱写了辉煌篇章，我们为自己取得的成绩而骄傲，我们也为周口的发展进步而自豪！

　　这五年，我们的综合实力大幅提升。全市经济总量实现由全省第九位到第五位的跃升。2015年生产总值达到2082.4亿元，连续三年稳居全省第五位，经济总量跻身全国城市前100名，居92位；人均生产总值由12934元提高到23644元，较“十一五”末增长1.7倍；一般公共预算收入突破100亿元，是“十一五”末的2.6倍；一般公共预算支出突破441亿元，是“十一五”末的2.3倍；规模以上工业增加值达到927.3亿元，是“十一五”末的2.1倍；社会消费品零售总额完成947.7亿元，是“十一五”末的2倍；固定资产投资累计完成5814.7亿元，是“十一五”期间的2.7倍；实际利用外资累计20.4亿美元，是“十一五”期间的4倍。

　　这五年，我们的粮食生产连创新高。粮食生产连续五年创历史新高，累计总产达到790亿斤，稳居全省第一位，周口市和7个县市连续荣获“全国粮食生产先进单位”。围绕建设现代农业强市，培育新型经营主体，发展适度规模经营，土地流转面积达到288.5万亩，市级以上农民示范合作社达到410家，工商登记家庭农场3375家，农业的信息化、标准化发展步伐加快，农业综合生产能力和现代化水平大幅提升。

　　这五年，我们的工业集聚亮点突出。工业始终是增长最快的产业，规模以上工业总数达到1204家，比“十一五”末增加110家，规模以上工业主营业务收入是“十一五”末的3.1倍。产业集聚区成为周口工业发展、城市壮大的有效载体，建成区面积达到166.8平方公里，累计完成投资3488.4亿元，拉动农村人口向城市集中100万人左右。沈丘产业集聚区被评为全省二星级产业集聚区，项城、太康、西华、郸城、商水、淮阳和周口经济技术产业集聚区被评为全省一星级产业集聚区，星级产业集聚区数量位居黄淮四市第一位。伴随着产业集聚区的发展，各县市立足实际，挖掘优势，奋勇争先，多数实现了在全省经济发展位次的前移，县域经济呈现蓬勃发展的局面。

　　这五年，我们的城市建设步伐加快。中心城区建设稳步推进，新老城区面貌发生较大改变，特别是2013年1月省政府批准设立了城乡一体化示范区，确立了周口新型城镇化的主攻方向，拉开了中心城区开发建设新一轮高潮，新区10余条主次干道全部畅通，五纵六横路网框架基本形成，市文化艺术中心、市体育中心、市行政中心、周口公园、和谐公园等项目相继建成，港口物流产业集聚区建设取得重大进展，华耀城、碧桂园、沙颍河生态建设等初具规模，城乡一体化示范区成为中心城区变化最大、最具发展活力的热土。鼓励支持县城加快产业发展，促进人口集聚，所有县城都已成为产城互动、秩序优良、充满生机、初具规模的现代中小城市。以重点镇建设为抓手，完善乡镇基础设施和公共服务设施，一部分重点城镇成为吸纳农村人口转移的重要载体。

　　这五年，我们的基础支撑不断壮大。五年中累计建设重点项目672个，其中建成投用497个，干成了一批打基础、利长远的大事，办妥了一批多年想办却办不了的难事、要事、好事，实现了一些具有标志意义的突破。产业合作领域得到拓展，实现了与美国好时、福喜集团合作，引进了富士康、娃哈哈等知名企业，吸引了鲁花、正大、大用、牧原、六和等企业在周口扎根落户。坚强电网建设迈出新步伐，实现了整体供电能力、110千伏以上变电容量、10千伏配电变压器容量“三个翻番”。天然气基础设施日趋完善，实现了管道天然气通达所有县市区。随着新周高速公路的开通，全市高速公路通车里程接近500公里，公路枢纽地位更加巩固。沙颍河通航将上溯至漯河，奠定了我市独特的水运优势。漯阜铁路改造升级全面完成，运力大大增强。公路、水路、铁路共同构成了周口大交通格局，周口融入全国、全省经济发展的基础更加坚实。

　　这五年，我们的民生福祉显著改善。坚持实施“十项重点民生工程”，持续抓好中心城区“十件民生实事”，财政民生支出累计达到1381.7亿元，占财政支出总量的79.1%。城镇居民人均可支配收入达到21019元，年均增长11.2%；农村居民人均可支配收入达到8576元，年均增长13.5%。群众就业、就医、住房、出行等条件不断改善，新型农村合作医疗、新农保实现了全覆盖，城镇就业累计新增44.3万人，农村劳动力转移就业累计新增53万人，解决386万农村居民和在校师生饮水安全问题，新建、改建农村公路3400公里、大中桥梁13500延米，90.5万农村贫困人口稳定脱贫，人民群众的获得感、幸福感不断增强。

　　这五年，我们的教育事业蓬勃发展。全市累计投入教育资金425.6亿元，促进各级各类教育快速协调发展，涌现出践行焦裕禄精神的好校长张伟、感动中国的教师李灵等一大批全国优秀典型，创造了全国有影响的郸城教育经验和郸城一高名校，高招录取人数和名校录取人数连年增长，累计20.8万人考入本科院校，272名被清华、北大录取，5名全省文理科状元出自周口，义务教育巩固率、高等教育毛入学率分别达到98%和41%，为国家培养和输送了人才，被国务院表彰为全国“两基”工作先进单位。教育为周口争得了荣誉，为周口未来的发展积蓄了宝贵资源。

　　这五年，我们的总体形象越来越好。在市委的坚强领导下，全市形成了积极向上、风清气正的良好政治生态，大家自觉维护来之不易的大好局面，各民族和睦相处，全社会安居乐业。同时，随着农业大市向经济大市的转变和综合经济实力的不断增强，周口人团结一致、务实重干、奋力拼搏、积极进取的形象正在不断形成和光大；大批外出创业成功人士活跃在全国各地，周口人勤劳善良，敬业奉献，能干事、干成事的形象更加出彩；郑春光、彭秀英、张伟等一大批英模人物的涌现和频频出镜于央视等主流媒体，周口人助人为乐、见义勇为、诚实守信、遵纪守法的形象备受社会点赞。周口的外在形象越来越好，周口人民的发展自信越来越强！

　　各位代表！

　　“十二五”的五年，是我们创造了辉煌的五年，也是我们挥洒汗水、砥砺奋进的五年。五年的成就来之不易，这是省委、省政府和市委正确领导的结果，是市人大及其常委会依法监督、大力支持的结果，是市政协积极建言献策、鼎力帮助的结果，是全市上下团结拼搏、克难攻坚的结果。在此，我代表市人民政府，向辛勤工作在各个领域的全市人民，向市人大代表、政协委员，向各民主党派、工商联、人民团体和离退休老同志，向驻周人民解放军、武警官兵、公安政法干警，向所有关心、支持、参与周口建设和发展的中央和省驻周单位、社会各界人士、海内外朋友，致以衷心的感谢和崇高的敬意！

　　各位代表！

　　成绩值得珍惜，问题不容回避，我们必须清醒忧患，充分认识到在实现周口崛起的道路上，还存在着许多困难和问题。从长期看，周口历史欠账多，发展相对落后的状况没有根本改变；经济结构不优，产业层次不高的状况没有根本改变；城镇化水平不高，中心城区首位度低的状况没有根本改变；公共服务能力不强，公共产品不能满足人民群众要求的状况没有根本改变；推动发展的能力不足，人才相对缺乏的状况没有根本改变。从短期看，一是我市经济运行质量效益不高的问题依然存在。规模以上工业企业培育数量不够，高新技术企业较少，企业创新能力、市场竞争能力不强，个别企业生产经营不景气，甚至出现停产半停产状况；房地产市场销售不旺，财政收入对房地产的依赖程度较大，工业对财政的贡献率相对不高，财源结构不够优化，财政收支矛盾突出。二是农民多而不富、农业大而不强、农村广而不美的问题依然存在。全市还有贫困村630个、贫困人口52.4万人，脱贫攻坚任务繁重；农业现代化水平不高，新型农业经营主体培育不足，在全国、全省有影响的农业品牌不多，与农业大市地位不相称；农村环境问题还很突出，垃圾围村、污水横流现象比较普遍，生产生活条件需要进一步改善。三是招商引资、项目建设后劲不足，推进不尽理想的问题依然存在。招商引资引进的集团型、基地型大项目少，一些项目效益不理想，合同履约率、项目落地率、资金到位率及项目层次都有待提升；一些重点项目建设进度缓慢，部分没有按时序节点推进，没有按时形成新的经济增长点。四是城镇化水平不高，中心城区发展滞后、辐射带动力弱的问题依然存在。城镇化发展速度和质量低于全省平均水平，城市对农村的拉动作用不强，尤其是中心城区综合经济实力弱，产业结构不合理，城市基础设施差，城市管理体制不完善，老城区改造进展较慢，市区内“脏、乱、差、堵”和“两违”现象没有得到根本遏制，市民对此意见还很大。五是政府系统作风不严不实的问题依然存在。通过开展群众路线教育实践活动、“三严三实”专题教育、懒政怠政为官不为专项治理和项目建设环境集中整治等活动，一些部门乱作为现象有所减少，但是个别干部缺乏开拓创新意识和责任担当，遇事畏首畏尾、推诿拖延，不作为、慢作为现象依然存在；个别单位工作人员尤其是中层干部，利用职权“吃拿卡要”的现象时有发生，发展环境有待进一步整治优化。对于这些问题，我们一定高度重视，不回避、不遮掩，采取得力措施，切实加以解决。

　　二、“十三五”时期主要目标和任务

　　“十三五”时期是全面建成小康社会的决胜阶段，也是我市追赶跨越的重要战略机遇期。根据市委建议，市政府编制了《周口市国民经济和社会发展第十三个五年规划纲要（草案）》，已印发会议，提请审议。

　　“十三五”时期经济社会发展的指导思想是：高举中国特色社会主义伟大旗帜，全面贯彻落实党的十八大和十八届三中、四中、五中全会精神，以马列主义、毛泽东思想、邓小平理论、“三个代表”重要思想、科学发展观为指导，深入贯彻习近平总书记系列重要讲话精神，按照“五位一体”总体布局和“四个全面”战略布局，坚定不移地实施周口崛起方略，坚持发展是第一要务，牢固树立创新、协调、绿色、开放、共享的发展理念，以提高经济发展质量和效益为中心，加快形成适应经济发展新常态的体制机制和发展方式，围绕“一极两区三中心”发展定位，积极融入国家“一带一路”战略和郑合高铁沿线经济发展轴带，着力构建“一中心四组团五支撑”发展格局，着力优化经济结构、转换发展动力，着力保障和改善民生，加快推进“四化”同步科学发展，确保如期全面建成小康社会，为全面实现周口崛起奠定坚实基础。

　　今后五年经济社会发展的总体目标是：经济保持较高速度增长，主要经济指标年均增速高于全省平均水平，缩小经济社会发展主要人均指标与全省平均水平差距，到2020年全市生产总值和城乡居民收入比2010年翻一番以上，力争实现“三个突破”：生产总值突破3500亿元，规模以上工业增加值突破1500亿元，一般公共预算收入突破160亿元，“一极两区三中心”地位更加突出，如期全面建成小康社会，周口崛起迈上新的台阶。

　　各位代表！

　　目标催人奋进，实干创造辉煌。“十三五”的五年，必将成为我们追赶跨越、争先晋位的关键五年。做好“十三五”的工作，对周口的发展至关重要，对周口的形象至关重要，对周口1100多万人民的幸福生活至关重要。我们必须把握新常态这个经济发展的大背景，应时合势，实干苦干，牢牢把握经济社会发展主动权。为此，必须始终坚持发展第一要务，努力促进经济实现跨越发展。加快跨越发展是全市人民的迫切追求，也是省委、省政府对周口提出的明确要求，我们必须毫不动摇地坚持以经济建设为中心，坚持把当前稳增长与长远保态势结合起来，把加快中心城区发展与促进县域经济发展结合起来，准确把握新常态带来的趋势性变化，遵循规律、大胆作为，保持定力、抓住关键，壮大总量、补齐短板，城乡一体、共进共荣，奋力开拓周口发展新境界。必须始终坚持五大发展理念，努力促进经济转型发展。牢固树立创新、协调、绿色、开放、共享发展理念，以发展理念转变引领发展方式转变，以发展方式转变推动经济转型升级，实现投资有回报、产品有市场、企业有利润、政府有税收、生态有改善、人民有获得感。必须始终坚持项目带动，努力促进投资持续较快增长。持续以项目建设为抓手，以“双十五”工程和中心城区“四个一批”项目为重点，坚持重点工作项目制、重点项目目标制、重要目标责任制，完善推进机制，强化责任落实，持续提高项目建设管理的精准性、有效性，壮大经济实力，保持发展活力。必须始终坚持统筹平衡，努力促进社会稳定和谐。始终保持清醒忧患，正确处理改革发展稳定的关系，防止各类风险的发生，提高安全生产和非法集资等风险的预警防控能力，创新社会治理，为改革发展创造良好环境。必须始终坚持务实重干，努力促进工作效能不断提升。继续按照“三严三实”的要求，发扬务实重干、勇于担当的作风，提升驾驭全局的能力，抓大事、干实事，研专题、出实招，坚决不走错路、少走弯路，坚决不务虚名、多求实功，全面提升工作效能。

　　各位代表！

　　只要我们坚持做到上述五个“始终坚持”，不动摇、不懈怠、不折腾，顽强奋斗、艰苦奋斗、不懈奋斗，拼搏进取、久久为功，我们就能不断开创周口发展的光明前景！

　　三、2016年目标任务和重点工作

　　2016年，我们将认真落实中央、省委、省政府和市委的各项决策部署，大力推进供给侧结构性改革，着力稳增长、促改革、调结构、强基础、惠民生、防风险、降成本、提效益，确保“十三五”良好开局。

　　今年经济社会发展的主要预期目标是：生产总值增长8.5%左右；地方一般公共预算收入增长8.5%；全社会固定资产投资增长15%；社会消费品零售总额增长12%；外贸进出口总额增长7%；实际利用外商直接投资增长5%；城乡居民收入与经济增长同步；城镇新增就业6.3万人以上；居民消费价格涨幅3%左右；节能减排、环保等约束性指标完成省定目标；发展质量和效益明显提高，结构调整和改革创新取得新的进展。

　　上述目标，总体高于全省平均水平。面对复杂严峻的经济形势，之所以这样确定，是因为综合分析判断，我市仍然具备加快发展的基础和条件。从发展基础上讲，近几年，我们深入实施周口崛起方略，全市追赶跨越的态势得到巩固，主要经济指标增速已连续4年高于全省平均水平，经济总量稳居全省第五位，经济大市的地位日益凸显。我们办成了一些大事要事，实施了一批对周口未来发展意义重大的项目，蓄积了更多崛起的基础和力量。从产业结构上讲，我市农业具有基础优势，现代农业发展潜力巨大。我市工业多数是农副产品加工业，去产能、去库存的压力小，具备适应市场需求、快捷利用创新技术调整产品结构的条件，在转方式、调结构的新形势下，工业增长已连续4年在全省保持领先位次。我市有一千多万人口，消费市场潜力巨大，以商贸物流为特色的服务业规模不断壮大，对经济增长的拉动作用日益增强。从资源禀赋上讲，我市人力资源丰富，在全国人口红利逐渐消退的背景下，我市的劳动力资源优势更加明显，加上每年有20万左右外出劳动力回乡创业就业，资金技术等生产要素加速回流，能够增强经济发展的活力和潜力。我市的生态环境优势较为明显，自源性污染少，水生态、林业生态均处于良好状态，成为新的竞争优势。从政策机遇上讲，我国发展仍处于可以大有作为的重要战略机遇期，机遇大于挑战。中央和省委、省政府为保持经济平稳健康发展，陆续提出了许多新理念、新战略、新举措，比如，着力推进农村一二三产业融合发展、提高基础设施网络化水平、重点支持中西部地区城镇化、实施脱贫攻坚计划等等，并配套出台了一系列政策措施，这些政策措施与我市当前发展的需求紧密契合，有利于我们争取更多政策资金支持。加上我们具有良好的政治生态，有对富民强市的不懈追求，有全市上下思发展、谋发展、促发展的高涨热情，有比干劲、比贡献、比速度的浓厚氛围，只要我们坚定信心，埋头苦干，我们一定能够全面完成今年的预定目标，一定能够在全面建成小康社会的道路上迈出坚实步伐。

　　为此，我们要努力做到“十个突出”“十个持续”。

　　（一）突出加强项目建设核心地位，持续增强经济发展后劲。强化项目带动战略，牢牢抓住项目建设这个总抓手，坚持稳定增长与培植后劲相统一，突出抓好事关经济长远发展、具有基础支撑和带动作用的重大项目。

　　深化项目服务保障。围绕以“双十五”和中心城区“四个一批”为核心的226个市级重点项目，集中人力、物力、财力，促开工、争进度，保投运、见效益，确保完成年度建设任务。建立健全重点项目工作台账，推行“模拟审批”制度，为项目实施创造条件。强化政府部门服务项目的意识，对重点建设项目，优先保障建设用地，优先保障环境准入，优先保障资源配置。

　　强化项目跟踪督导。对重点项目实行动态管理，完善进入和退出机制，项目用地、财政补助、税收及行政性收费减免，与项目的形象进度、时序进度和投资额度挂钩，对不符合重点项目管理要求的，及时对重点项目库进行调整。加强项目督导检查，进一步提高项目建设的速度和质量。

　　积极谋划储备项目。认真研究国家、省有关投资政策、产业政策、区域经济政策，找准我市与国家和省战略规划的对接点，与国家部委和省直厅局主动对接，在基础设施、公共服务、产业转型升级、企业技术改造、现代信息技术融合运用等方面，筛选和推介一批重大项目。提高项目谋划质量，增加新开工项目比重、工业项目比重、中心城区项目比重、重点项目在固定资产投资中的比重，努力实现由单个项目谋划向产业链和产业集群谋划转变，争取有更多项目进入国家和省重点项目“盘子”。

　　（二）突出发挥工业主导作用，持续提升制造业实力。坚持工业兴市战略不动摇，抓住《中国制造2025》和“互联网＋”行动计划政策机遇，集中力量抓工业、强工业，提升工业综合实力。

　　加快产业集聚区提质转型创新发展。坚持“五规合一”、“四集一转”、产城互动的基本要求，突出集群、创新、智慧、绿色发展方向，继续推动产业集聚区上规模、上水平、上层次，打造县域经济发展主引擎。完成产业集聚区与乡镇行政区域管理体制套合。完善产业集聚区考核评价办法，严格以增加值、税收收入、从业人员、所在城镇城区常住人口为评价指标，提高考核评价的科学性、激励性。高度重视规模以上工业企业和“限上”服务企业培育，形成一批新的经济增长点。支持各县市区加快发展速度，壮大县域经济总量，强化争先晋位意识，实现在全省位次前移。

　　大力推进产业集群培育。突出链式发展和群体扩大，支持本土企业成长，积极承接产业转移，继续做大做强传统支柱产业集群。围绕面制品、肉制品、油脂深加工，开发健康、营养、保健、方便、休闲食品，促进食品加工业向价值链高端延伸，在千亿级产业集群培育上迈出新步伐。积极改造提升棉纺织业，提高精梳比重，发展高水平染整、中高档面料和高档服装、家用纺织品等终端产业，加快形成千亿级纺织服装产业集群。抓住国务院和省政府支持医药化工产业结构调整的政策机遇，完善医药产业链条，着力打造医药化工千亿级产业集群。继续培育电缆电气、装备制造、新型建材3个500亿级新兴产业集群，壮大制造业规模，提升产业竞争力。

　　着力推进企业转型升级。制订工业转型升级行动计划，推进传统产业高端化、新兴产业集群化、高新产业规模化。继续实施技术创新、质量提升和品牌建设，争取认定一批省级技术创新示范企业、省级工业公共研发设计中心和工业新产品。谋划、筛选一批重点技术改造项目，支持重点企业实施技术改造。加快企业战略重组，以30户重点企业和30户高成长企业为载体，鼓励有实力的企业以资产、技术和品牌为纽带，跨地区、跨行业兼并重组，培育竞争力强的大企业、大集团。加强上市后备企业培训，力争更多企业挂牌上市。稳妥化解过剩产能，扩大有效产能。继续开展市长质量奖评选，增强企业质量意识。淘汰不符合能耗、环保、质量、安全等标准的企业，处置“僵尸企业”，促进集约发展、绿色发展。

　　推进工业化与信息化融合发展。顺应国家建设制造业强国的战略部署，把智能制造作为“两化”融合发展的重要方向，鼓励采用智能制造装备技术，加强网络化制造技术、云计算、大数据在制造业中的深度运用，推动制造业向自动化、智能化转变。吸收和引进各类资本，推动建立“互联网＋”创业园和孵化器，建设周口“创客”队伍。大力发展生产性服务业，培育和引进供应链集成服务企业，拓展企业获取技术创新、管理创新成果的渠道，推动我市工业强筋骨、上水平。

　　优化企业服务保障。更加注重运用市场化、法治化手段，支持产业、企业发展。鼓励引导金融机构向工业企业提供融资支持，统筹做好电力、燃气、劳动力及土地等生产要素保障。抓好国家各项惠企政策落实，清理和规范涉企收费项目，优化企业发展环境。加强对企业经营者的培训，壮大我市优秀企业家队伍。鼓励引导企业培养优秀技工，弘扬工匠精神，建设周口本土工匠队伍。

　　（三）突出夯实农业发展基础，持续加快农业现代化步伐。牢记保障国家粮食安全和脱贫攻坚的政治责任，促进农村一二三产业融合发展，提高农业发展的质量效益，推进现代农业强市建设。

　　提高粮食综合生产能力。落实农业“三项补贴”改革，加大涉农资金整合力度，统筹推进水、电、路、林等农田基础设施建设，力争新建高标准粮田135万亩。继续开展“红旗渠”精神杯和“丰产杯”竞赛活动，进一步改善农田水利条件。抓好整乡建制的高产创建，力争每个县市建设3～5个整建制试点乡镇，所在乡镇分别建立2个小麦和2个玉米高产创建万亩示范方。划定永久基本农田，实行最严格的耕地保护政策，筑牢“藏粮于地”的基础。加强农业科技创新，推广运用农业新品种和农业新装备，提高农业综合生产能力，积蓄“藏粮于技”的潜力。

　　加快现代农业体系建设。持续推进农业发展方式转变，加快土地流转步伐，发展多种形式适度规模经营，鼓励种养结合、农牧一体、农林一体生态循环发展。引导金融机构加大对新型农业经营主体的支持力度，培育壮大以家庭农场、专业大户、专业合作社为主的新型农业经营主体。调整优化农业结构，促进农业生产经营专业化、规模化、集约化，提高土地产出率、资源利用率和农业劳动生产率，增强农业抗风险能力、市场竞争能力和可持续发展能力。鼓励引导新型经营主体采用农产品质量标准和质量安全追溯技术，深入推进农业生产标准化、信息化。加强农产品品牌培育，打造一批知名品牌。推进“互联网＋农业”，增强农业信息服务能力，力争所有县市区都与大型互联网企业建立合作关系，推动周口农业融入全国、走向世界。加强新型职业农民培训，培养新型农民企业家和专业能手队伍。

　　推动农村产业融合发展。落实国家有关促进农村产业融合发展的政策措施，积极推进省级农村一二三产业融合试点工作。树立大农业、大食物观念，继续推进现代农业产业化集群培育工程和都市生态农业工程，打造农产品精深加工链条、资源循环利用链条，促进农产品加工业与农田、养殖场、林场连接融合，推进农业产业链和价值链建设。积极培育农业技术推广、农田管护、农产品收储、流通等农业公共服务和社会化服务体系。加强农业多功能开发，积极发展休闲观光、创意农业等新型业态。

　　改善农村生产生活条件。着力推进社会主义新农村建设，加快城镇基础设施和公共服务设施向农村延伸，有计划地推进国道、省道改造升级，完成农村公路“三年行动计划”乡村畅通工程，年内新建农村公路1100公里，改造大中桥梁3000延米。加强农村危房改造。着力改善农村人居环境，以县级统筹为重点，整合农村人居环境整治等项目资金，解决垃圾围村、污水乱排问题，力争每个村建立1个垃圾集中收储堆放点，逐步建立农村垃圾收集、转运、处理机制。开展农村人居环境示范创建活动，建成一批示范村、示范乡（镇）、示范县，推动农村人居环境进一步改善。

　　全面推进脱贫攻坚。利用国家加大对贫困地区投入力度、改变贫困地区基础设施落后状况的各项政策，谋划、争取、实施一批农村基础设施项目、公共服务项目和产业扶持项目。启动三年脱贫攻坚计划，围绕2018年完成脱贫攻坚任务的要求，对贫困县和面上县实施分期脱贫管理，川汇区、东新区年内完成脱贫目标。切实做到扶贫对象精准、项目安排精准、资金使用精准、措施到户精准、因村派人精准、脱贫成效精准，力争实现稳定脱贫16.3万人。

　　（四）突出打造豫东南商贸物流中心，持续推进服务业快速发展。强化商贸物流传统优势，鼓励业态创新，完善发展载体，保持服务业快速成长势头，着力打造豫东南区域商贸物流中心。

　　加快发展商贸物流业。发挥区位交通优势、商贸物流业基础优势，推动商贸物流业品牌化、连锁化、集团化。加快华耀城二期工程、龙都红星广场项目建设，促进商贸物流企业加快集聚，扩大品牌影响，提升辐射带动能力。加快推进中原鞋城一期工程建设，尽早形成中心城区新的商贸繁荣区。理顺商务中心区建设管理体制，全面抓好商务中心区和特色商业区建设，尽快完善设施，培育产业，拓展服务业发展空间。

推动房地产业健康发展。把鼓励住房消费、加快消化库存作为促进房地产业发展的首要任务，支持缴存职工用公积金贷款改善住房条件，将有稳定劳动关系的常住人口纳入公积金覆盖范围，促进新市民在城镇购房，鼓励农民进城购房。建立购租并举住房制度，提高棚户区改造货币化安置率，培育发展住房租赁市场，公租房新增需求主要通过发放货币化补贴、政府购买或长期租赁商品房解决，推进房地产业健康稳定发展。引导房地产业发展养老、文化、体育等跨界地产，实现转型发展。规范物业管理市场，建设一批和谐精品社区，提高房地产市场品位。

继续壮大金融业。加快农信社组建农商行步伐，确保4家完成组建、5家达到组建标准。大力引进股份制银行、保险、证券等金融机构在我市设立分支机构，持续培育新兴金融业态，壮大金融业规模。加强社会诚信建设，加大对担保公司、小额贷款公司、投资类企业、典当行等机构的监管，优化金融生态，防范金融风险。

　　着力发展新兴服务业。加快发展科技研发、信息咨询、交通物流、检验检测等生产性服务业。大力发展文化旅游产业，全面做好文化与旅游的有机结合，丰富旅游业内涵，促进文化旅游产业融合发展。加快淮阳太昊陵、西华女娲城、项城袁氏故居、川汇区关帝庙等景区建设，完善旅游设施，提升旅游接待水平。积极发展楼宇经济、教育培训、人力资源服务、体育、信息服务等生活性服务业，重点支持健康养老产业，推动生活性服务业向精细化和高品质提升。利用“互联网＋”，推动在线教育、在线医疗等新业态快速发展。以电子商务为牵引，大力发展平台经济，支持建设电商产业园，继续推进与阿里巴巴集团的战略合作，组建和做大阿里巴巴周口产业带。打造“双创”示范基地，以周口师范学院大学生创业园为依托，逐步构建周口众创、众包、众扶、众筹网络支撑平台。

　　（五）突出提升中心城区引领作用，持续培育辐射带动能力。认真贯彻落实中央城市工作会议精神，坚持中心城区带动战略，以“五城联创”工作为抓手，统筹推进新城区开发和老城区改造，提高城市建设管理水平，培育辐射引领能力。

　　强化规划引领。坚持以人为本，创新规划理念和方法，增强规划的科学性和指导性，年内完成《周口市城市总体规划（2014～2030）》向省政府报批工作。完善中心城区水系规划编制、商务中心区和特色商业区规划编制。启动城市地下管网、城市综合管廊、城市综合交通、城市公交、城市停车设施、给排水、商业设施、园林绿化、海绵城市等专项规划编制。严格执行各类规划，提高规划的公开性、严肃性、强制性，一张蓝图绘到底。坚持多规合一，突出控制性规划的统筹性和精细性，将各类设施一次性规划、高标准建设，避免前建后拆，提升城市建设的质量和品位。

　　加快设施建设。紧紧抓住国家支持中西部城市建设的政策机遇，加快中心城区各类设施建设，提升城市综合承载能力，促进城市面貌尽快改善。继续改善4个高速公路出入口总体形象，年内完成汉阳北路、太清路、文昌大道东段路面提升改造、新周高速连接线二期等续建项目。积极谋划融入郑合高铁经济轴带，高标准做好周口高铁站片区的规划设计和开发建设前期工作。提升改造一批城市主干道。启动中原路打通和中原路跨沙颍河大桥工程、开元大道东延与东外环路连接工程、新火车站配套路网南八路、黄河路与中原路打通工程等。继续推进沙颍河周口至省界航道升级改建工程和港区港口中作业区码头建设。完善城市公共交通体系，开工建设华耀城客运站，加快建设周口客运西站，推进绿色低碳交通基础设施建设。继续改造自来水供水管网，完成老自来水厂与南水北调管网连接，建成投用南水北调东区水厂，尽早让市民吃上干净卫生的丹江水。启动建筑垃圾处理厂项目。加快建设污泥处置和污水处理项目，继续改造城区污水管网。建设隆达电厂中水回用项目。建成周西500千伏变电站。继续改造建设一批新的背街小巷、停车场、健身广场（园）、垃圾中转站、公共厕所。推进周口至南阳高速公路项目前期，力争年内开工建设。启动周口东客运枢纽、大闸桥拓宽工程、北环路工程前期工作。

　　完善城市功能。坚持新老城区并重，加快完善城市公共服务功能，提升人口集聚能力。在城乡一体化示范区实施精品片区建设，重点围绕综合服务功能区，谋划和引进教育、医疗、养老、商贸等项目，打造城市新亮点。启动原荷花市场及周边改造开发，建设高端城市综合体。加快推进东成棚户区改造工程建设，改善七一路东段区域城市形象。建成并开放沙颍河公园（周口大道至东外环路段）。启动流沙河、洼冲沟等6条河道中心城区段生态水系治理工程和引黄调蓄工程建设，推进园林城市建设，增强城市魅力。

　　推进精细管理。认真贯彻中央关于推进城市执法体制改革改进城市管理工作的指导意见，启动城市管理数字化平台建设，继续推进中心城区城市管理向长效化、精细化、规范化转变。持续开展“脏、乱、差、堵”综合治理，加快卫生城市、文明城市建设步伐。保持“两违”治理高压态势，杜绝“两违”增量，让违法建筑者得不偿失、付出代价，直至绳之以法。严格城市工程建设质量管理，设置市政工程建设铭牌，公开施工方和监管方信息，实行质量责任终身追究。继续推进精品街区建设工程，再建一批严管街、示范路、高品质社区。

　　壮大产业实力。下大力气加快中心城区产业发展，力争主要经济指标高于全市平均水平。依托经济开发区、高新技术产业开发区、港口物流产业集聚区，集中力量抓好世通巴铁等一批重大工业项目，壮大中心城区工业规模，提升中心城区工业实力。统筹各产业集聚区的主导产业，促进同类产业的集群集聚布局，避免无序竞争。加快商务中心区、华耀城二期工程的开发建设和老城区传统商贸圈的规范提升，增添中心城区商贸物流业发展的人气和活力。建设1～2个都市生态农业、休闲农业园区，发展都市农业旅游。利用大数据技术，建立中心城区统一的产业数据分析平台，研判中心城区产业发展态势，提升决策的预见性、准确性。

　　促进联动发展。围绕“一中心、四组团、五支撑”发展格局，坚持中心带动、联动发展，继续推进中心城区与项城、淮阳、商水、西华融合对接，启动实施八一路连通商水工程，加快周项快速通道建设，实现各组团县城与中心城区优势互补、良性互动。支持组团和支撑县城，完善基础设施，壮大产业规模，提高人口吸纳和承载能力，建设富有活力的中小城市。配合省政府确定的重点镇建设，各县市要选择一批基础较好的重点镇，加强规划建设，进一步完善城镇体系。落实户籍改革和居住证制度改革政策，推动农业转移人口市民化，稳步提升全市城镇化水平。

　　（六）突出用好开放招商关键举措，持续增强经济发展活力。顺应开放新形势，开展“招商引资突破年”活动，着力引进一批优质项目，为经济社会发展注入新的动力。

　　开展“招商引资突破年”活动。实施开放招商“一把手”负责制，筛选全国知名企业，实行部门分包联系责任制，做好牵线、搭桥、引进的各项工作。各县市区、市直各经济主管部门要把招商引资工作放在突出位置，确定年度引资目标、落地项目总数和投资总额，加强组织领导，跟踪问效落实。积极主动跟踪洽谈好中原国际智能养老健康城项目、乡村旅游项目、华侨医院项目等重点招商项目，力争年内落地一批具有引领性、突破性、方向性的重大项目，形成一批新的经济增长点。

　　创新招商方式。继续实行产业链招商、集群招商等有效招商方式，更加注重紧盯重点区域和重点对象实施精准招商，更加注重以商招商，更加注重现有企业增资扩能实现“零地招商”，更加注重吸引在外周口人回乡投资创业。继续组织专业招商队伍，专门研判我市企业发展的需求、外地产业资本的投资动态，实现招商引资主动式对接，提高招商引资的精准性。

　　培育开放招商优势。积极对接郑州航空港经济综合实验区，支持有条件的县市建设海关特殊监管区和保税中心。配合中原鞋城建设，充分利用广东鞋业厂商会在制鞋行业的影响，引进一批品牌鞋业生产企业，加快建设制鞋综合检测中心，巩固我市作为河南制鞋产业出口基地的优势。强化服务措施，建立谈判、签约、落地、建设、投产、壮大等全过程服务链，提高招商引资项目的履约率、开工率、资金到位率、投产达产率。继续实行首问负责制、服务承诺制、限时办结制、无偿代理制，完善一站式、一条龙服务，营造亲商、安商氛围，维护周口良好的开放形象。鼓励本土企业开展国际合作，不断扩大利用外资规模，提高进出口贸易的质量和效益。挖掘我市清真食品、用品生产优势，谋划建设清真食品产业园，推动产品走向国际市场，拓展企业发展空间。

　　（七）突出深化改革创新发展，持续释放经济发展新动能。坚持用改革激发活力，用创新提供动力，完成好各项重大改革任务，释放改革红利，推进创新创业。

　　深化行政体制改革。推进政府职能转变，提高服务的质量和效率。围绕简政放权，继续取消、下放行政审批事项。清理和规范中介服务事项，坚决切割审批部门与中介机构的利益关系。尽量减少不必要的评审、核查，减轻企业负担。推行“互联网＋政务服务”，逐步将所有行政审批事项纳入网上审批。全面建立权责清单制度，健全运行机制，推进依法行政。注重加强日常监管，做到监管常态化、制度化，提高监管的有效性，逐步解决“只重审批、不重监管、批后不管”的问题。加强公共资源交易平台建设，适当分流交易项目，提高交易效率，规范交易活动，加强交易监管，防止腐败滋长。深化商事制度改革，巩固扩大“三证合一”改革成果。鼓励复员退伍军人、高校毕业生和返乡人员在家乡创业兴业，让能者有所为，勤者有所获。全面完成政府机构改革、公务用车制度改革，持续推进事业单位改革，深化财税体制改革，统筹抓好价格、教育、文化等领域的改革。

　　深化科技和投融资体制改革。认真贯彻落实中央和省有关科技创新体制改革精神，落实国家有关科技成果转化政策，激励科技人员主动转化创新成果，促进创新技术转化为生产能力。有效整合各领域的创新力量，加大科技投入，不断满足经济社会发展对创新成果的需求。深化与省科技厅的合作，抓好议定事项落实。全面贯彻落实省政府企业投资项目负面清单制度，杜绝禁止类和限制类项目落地。利用好政府和社会资本合作（PPP）融资模式，组织好国家专项基金项目的申报争取工作。用好地方政府发债政策，提高发展资金保障能力。

　　深入推进创新发展。依托市内国家级、省级研发中心和工程技术中心，争取上级科研专项资金，努力突破一批产业关键技术。实施一批科技创新专项，重点抓好“医药中间体生产工艺技术研究及产业化”等4项省级科技专项的实施。全面提升市农科院科技创新能力，保持小麦、大豆育种国内领先优势。充分用好周口师范学院科研资源，鼓励实施科技创新攻关。加快郸城县国家级农业科技园区建设，推动周口高新技术产业开发区发展，培育壮大我市高新技术企业群体，提升创新发展能力。

　　（八）突出构筑绿色发展屏障，持续建设美丽周口。牢固树立绿色发展理念，把生态环境建设放在更加重要的位置，涵养和巩固生态优势，建设蓝天碧水美丽周口。

　　硬起手腕治理环境。继续实施蓝天工程，做好工业大气污染防治、燃煤锅炉拆改，加强机动车污染防治，加大城市施工和道路扬尘治理，提高空气质量。继续实施碧水工程，落实重点河流环境综合整治和最严格的水资源保护制度，加快推进中心城区水系治理工程，加大洺河等重点流域水污染防治。协调督促贾鲁河上游城市污水治理和减排，提高过境水质。加强污水排放和污水处理设施运行监管，确保饮用水水源地取水水质达标率100%。继续开展乡村清洁工程，推进农业面源污染治理。

　　狠抓源头污染防控。坚决杜绝新建污染型企业，淘汰或转型升级污染型企业。严控农作物秸秆焚烧，加大秸秆还田奖励补贴力度，探索秸秆综合利用新途径，促进农作物秸秆全量化综合利用。加强节水节能宣传，加强排污企业在线监管，严惩偷排超排行为。全面完成省政府下达的节能减排任务，减少资源消耗。

　　加强林业生态建设。继续推进全国绿化模范城市创建工作，年内新增造林绿化面积10万亩以上。加快实施生态廊道网络建设工程，廊道绿化率达到95%以上。继续实施“百千万”农田防护林建设工程，农田林网控制率达到95%以上。建成一批林业生态示范乡（镇、办）、示范村。大力实施城市绿化美化工程，提高城市绿化覆盖率、森林覆盖率，建设生态、宜居、美丽新周口。

　　（九）突出加强精神文明建设，持续促进周口文化繁荣。以社会主义核心价值观教育为引领，以文明周口建设为抓手，不断满足人民群众的精神文化需求，全面促进文化繁荣，提高文化供给能力。

　　提升文明素质。加强诚信建设，弘扬公序良俗，强化规则意识，传承良好家风，提升市民道德文化素质。继续开展“出彩周口人”系列报道，讲好周口故事，树立良好对外形象。大力宣传推广城市精神和市标、市歌、市树、市花等文化符号，涵养城市文化，提高居民对城市的认同感、归属感、自豪感。

　　加强文化供给。以满足市民多样化的文化需求为重点，实施文化惠民工程，推进公共文化服务设施建设，坚持免费开放。倡导全民阅读，建设书香社会。为市图书馆再购图书5万册，推进周口市数字图书馆工程。组织实施好中原文化大舞台惠民演出活动。组织好第七届中原古韵——中国（淮阳）非物质文化遗产展演活动。建设周口科技馆二期工程。加强网上思想文化阵地建设，净化网络空间。开展“扫黄打非”，维护文化安全。

　　繁荣文化产业。推进文化与科技、金融、旅游、互联网融合发展，传统媒体与新兴媒体融合互动。实施文化品牌战略，挖掘伏羲文化、女娲创世文化等丰富文化资源，拓展文化产业发展融资平台，破解文化产业发展资金瓶颈。推进广电等传统媒体与新兴媒体融合，打造全媒体文化平台，推动文化企业规模化、品牌化。加强文物资源的开发与保护。引进一批文化旅游项目，壮大文化旅游产业规模。充分发挥书画、戏曲、杂技等文化资源优势，支持民办文化场馆建设，鼓励文化企业走出去，拓展发展空间。继续支持淮阳文化改革发展试验区建设。

　　（十）突出强化共享发展理念，持续提高民生福祉。以十项重点民生工程和中心城区十件民生实事为抓手，切实解决人民群众最关心、最直接、最现实的民生问题，让人民群众共享改革发展成果。

　　加强就业和社会保障工作。健全城乡公共就业服务体系，继续实施以创业带动就业工程和全民技能振兴工程，做好高校毕业生和农村转移劳动力、城镇困难人员等群体就业工作，提高劳动者素质和就业质量。年内新增失业人员再就业2.3万人，就业困难人员实现就业9000人。加快城乡社会保障体系建设，依法扩大社会保险覆盖面。稳妥推进机关事业单位养老保险制度改革，做好新老制度的衔接和并轨工作。统筹推进城乡救助体系建设，完善最低生活保障、特困人员供养、困难群体重特大疾病医疗救助、残疾人兜底等制度。积极做好养老事业，加快中心城区养老服务示范点和市老年护理院二期工程建设。推进保障性安居工程、城中村（棚户区）改造建设，不断改善低收入群体居住条件。

　　办好人民满意的教育。以构建周口“大教育”为抓手，着力推进义务教育均衡发展，年内郸城县、扶沟县通过省达标验收。加快标准化学校建设，实施农村薄弱学校改造工程和扩充城镇义务教育资源五年计划。巩固高中教育质量，打造周口教育名片。持之以恒推进中心城区教育振兴工程。继续实施职业教育攻坚二期工程、学前教育三年行动计划二期工程，支持周口科技职业学院晋升本科、联营职专晋升大专，鼓励周口职业技术学院、周口幼师和周口技工学校等加快升级发展。继续支持周口师范学院建设河南省示范性应用型高校。大力发展民办教育，鼓励和吸引社会资金发展教育事业。落实好乡村教师支持计划。完善助学资助体系，加大对经济困难学生的帮扶力度。

　　发展卫生健康事业。深化医药卫生体制改革，抓好县级公立医院综合改革，启动城市公立医院综合改革试点，强化基层卫生服务能力建设，提升基本公共卫生服务均等化水平。加强公共卫生和重大疾病预防控制，大力促进中医药事业和健康服务业发展，广泛开展爱国卫生运动。推进市中心医院新区分院和市中医院新区分院建设。完善食品药品安全监管体制机制，加大社会共治力度，让人民群众吃得放心、吃得健康。全面实施“两孩”政策，提高生育服务水平。落实国家全民健身计划，办好市第三届运动会，加快市游泳馆、重竞技馆、老年人健身馆及各县市体育中心建设，完善全民健身服务设施，全力备战省第十三届运动会。

　　维护社会大局和谐稳定。畅通人民群众利益诉求渠道，充分发挥市长热线联系群众、畅通民意的作用，及时为民排忧解难。依法规范信访，落实逐级走访，加强舆情研判，重点解决好劳资纠纷、环境污染、征地拆迁、非法集资等突出问题，维护好群众利益。着眼“双安”，推进“双治”，强化“双基”，抓好社会治安综合治理，完善治安防控体系，依法打击各种违法犯罪行为，增强人民群众安全感。加强重大公共安全事件防范、预警、应急、救援体系建设，提高预防和处置突发事件的能力。落实安全生产责任，确保不发生重特大安全事故，切实维护社会大局和谐稳定。

　　继续开展双拥共建，积极创建双拥模范城，支持驻周解放军、武警部队、预备役部队、民兵和人民防空建设，促进军民融合深度发展。维护农民工、妇女儿童合法权益，关心残疾人事业。加强民族宗教工作，促进民族经济发展和民族团结进步。支持工会、共青团、妇联、工商联等人民团体在经济社会发展中发挥桥梁纽带作用。继续做好统计、物价、海关、气象、地震、史志、外事、侨务、社会科学、应急救援、对口援疆等工作。

　　四、勇于担当，主动作为，不断提升政府治理能力和水平

　　坚持忠诚于宪法，忠实于人民，着力建设法治政府、担当政府、服务政府、廉洁政府，不断提高治理能力，推进周口发展，造福全市人民。

　　始终坚定正确政治方向。巩固拓展群众路线教育实践活动和“三严三实”专题教育成果，认真抓好“学党章党规、学系列讲话、做合格党员”学习教育，严明政治纪律，严守政治规矩，坚决在思想上政治上行动上与以习近平同志为总书记的党中央保持高度一致。坚决服从市委统一领导，坚决贯彻市委决策部署，全力维护全市工作大局、发展大局。

　　毫不动摇坚持依法行政。贯彻国家法治政府建设实施纲要，启动“七五”普法，完善国家工作人员尊法学法守法用法制度，建立健全政府法律顾问制度，提高依法行政的能力和水平。自觉接受人大及其常委会的法律监督和工作监督，坚决执行市人大及其常委会的决议和决定，坚持重大事项报告制度；自觉接受人民政协的民主监督，密切与政协委员、各民主党派的民主协商，认真办理人大代表建议和政协提案。积极配合人大做好地方立法工作。全面推进政务公开，落实行政执法责任，加强行政执法监督，坚决纠正执法不公正、不文明现象。

　　持之以恒强化责任担当。继续弘扬敢于负责、勇于担当、务实重干的精神，以“懒政怠政为官不为问责年”活动为载体，解决“能力不足不能为、动力不足不想为、担当不足不敢为”和“吃拿卡要”乱作为等问题，重点查处机关中层干部执行不力、失职渎职、弄虚作假和态度蛮横、作风敷衍、故意刁难等影响经济发展和政府形象的人和事，下决心打通政策落实中的“中梗阻”和“最后一公里”。坚决惩治阳奉阴违、有令不行、有禁不止的行为，确保政令畅通，促使各级各部门一心为公、勤政为民，想干事、能干事、干成事，对得起肩上的使命，对得起人民的期待。

　　驰而不息加强廉政建设。严格落实党风廉政建设主体责任和“一岗双责”，深入推进党风廉政建设和反腐败斗争。严格执行中央八项规定和省委、省政府20条意见，持续深入反对“四风”，坚决惩治和查处损害群众利益的不正之风和腐败问题。认真抓好巡视反馈问题的整改落实，坚持把巡视工作的成果落实到加快发展上来。严格落实国务院“约法三章”，厉行节约，勤俭办一切事业。坚持用制度管权、管事、管人，加大对领导干部经济责任审计力度，推动审计监督全覆盖，让权力在阳光下运行。

　　各位代表！

　　政府的宗旨是为人民服务，政府的责任是推进经济社会更好更快的发展。踏着“十二五”圆满收官的鼓点，我们迎来了充满希望的“十三五”开局之年，踏上了推进周口发展的崭新征程，让我们更加紧密地团结在以习近平同志为总书记的党中央周围，在省委、省政府和市委的正确领导下，以滚石上山的精神和毅力，以抓铁有痕、踏石留印的劲头和作风，不惧矛盾，不畏艰险，牢记使命，负重前行，不断开创周口崛起、富民强市、全面建成小康社会的新局面！
